# Newborn Hearing Screening Benefits Children, But Global Disparities Persist

**DOI:** 10.3390/jcm11010271

**Published:** 2022-01-05

**Authors:** Katrin Neumann, Philipp Mathmann, Shelly Chadha, Harald A. Euler, Karl R. White

**Affiliations:** 1 Department of Phoniatrics and Pedaudiology, University Hospital Münster, 48149 Münster, Germany; Philipp.Mathmann@ukmuenster.de (P.M.); euler@uni-kassel.de (H.A.E.); 2 Blindness Deafness Prevention, Disability and Rehabilitation Unit, Department for Management of Noncommunicable Diseases, Disability, Violence and Injury Prevention, World Health Organization, 1211 Geneva, Switzerland; chadhas@who.int; 3 National Center for Hearing Assessment and Management, Utah State University, Logan, UT 84322, USA; Karl.White@usu.edu

**Keywords:** newborn, neonatal, hearing, screening, universal, NHS, infant, hearing loss, WHO, world report on hearing

## Abstract

There is substantial evidence that newborn hearing screening (NHS) reduces the negative sequelae of permanent childhood hearing loss (PCHL) if performed in programs that aim to screen all newborns in a region or nation (often referred to as Universal Newborn Hearing Screening or UNHS). The World Health Organization (WHO) has called in two resolutions for the implementation of such programs and for the collection of large-scale data. To assess the global status of NHS programs we surveyed individuals potentially involved with newborn and infant hearing screening (NIHS) in 196 countries/territories (in the following text referred to as countries). Replies were returned from 158 countries. The results indicated that 38% of the world’s newborns and infants had no or minimal hearing screening and 33% screened at least 85% of the babies (hereafter referred to as UNHS). Hearing screening programs varied considerably in quality, data acquisition, and accessibility of services for children with PCHL. In this article, we summarize the main results of the survey in the context of several recent WHO publications, particularly the World Report on Hearing, which defined advances in the implementation of NHS programs in the Member States as one of three key indicators of worldwide progress in ear and hearing care (EHC).

## 1. Introduction

Unaddressed permanent hearing loss, particularly when it is congenital or acquired early in life, significantly impedes a child’s development [1,2]. Permanent childhood hearing loss (PCHL) is associated with deficits in language, cognitive, psychosocial, educational, and vocational development, as well as with negative effects on employment and earnings [2,3]. There is overwhelming evidence that newborn hearing screening (NHS) significantly reduces the age of diagnosis and intervention of PCHL and that hearing-impaired children who were identified early through NHS and received timely diagnosis and suitable rehabilitation with hearing aids [4] or cochlear implants [5] or who participated in early intervention services [6], perform better in their overall language development [7,8,9], vocabulary [10], other developmental scores, and quality of life [11] than children without hearing screening. If babies with PCHL are enrolled in intervention programs within their first few months of life they even can achieve language and socioemotional developmental trajectories corresponding to their chronological age [2,6,9,12,13,14,15,16,17,18]. This requires, however, strong tracking and follow-up procedures subsequent to the NHS [19]. Positive long-term effects of NHS have been demonstrated in several large studies. For example, in the Australian LOCHI (Longitudinal Outcomes of Children with Hearing Impairment) study the provision of hearing devices as early as possible to children who were deaf or hard of hearing led to improved language performance over time [7,20]. In another study, teenagers from a birth cohort of 157,000 children from southern England who had received universal newborn hearing screening (UNHS) showed better reading comprehension than a control group from the same cohort who had not received NHS [21].

Some potentially negative consequences of NHS programs need to be considered such as parental uncertainty about screening results and further optimal diagnostic and treatment pathways for their baby when very young [22], but parental perspective studies have demonstrated that the benefits of early identification and intervention (EHDI) outweigh the aforementioned disadvantages [23,24,25,26].

Cost-effectiveness of NHS has been demonstrated [2,8,27,28]. In 2016, WHO published that more than 60% of hearing loss could be prevented—75% in middle- and lower-middle-income countries, 46% in high-income regions [3]. UNHS and prevention have been shown to be most effective in reducing the prevalence of PCHL and its sequelae, with UNHS being very effective for high-income countries, and prevention expected to show higher relative effects for low-income countries [29].

As far back as 1995, a WHO resolution was adopted that urged member states to prepare national plans for the prevention and control of major causes of avoidable hearing loss and for early detection of hearing loss in babies, toddlers, and children [30]. Yet, in 2012, only 32 countries had reported the implementation of such policies, and the WHO deplored a scarcity of epidemiological and other data regarding ear and hearing care (EHC) [31]. A second WHO resolution, adopted in 2017, reaffirmed the aims of the first and called on member states to collect high-quality population-based data on hearing loss and ear diseases [32].

Consistent with this goal and as a basis for further improving the effectiveness of NHS programs, a survey was recently conducted on the global status of program coverage, strategies, and outcomes, as well as the relationship between national economic indicators and key screening metrics of newborn and infant hearing screening (NIHS) [33]. Hearing screening of infants up to the end of the first year of life was included, as some programs screen babies later, e.g., as part of immunization programs, when they are no longer newborns.

In response to the 2017 resolution, the WHO released the first World Report on Hearing in March 2021 [2]. This report summarized epidemiological and financial data on hearing loss around the world and proposed cost-effective solutions for achieving “integrated people-centered ear and hearing care” (IPC-EHC). The report recognized NHS as playing a key role in IPC-EHC and identified “effective coverage of newborn hearing screening services within the population” (the proportion of infants with PCHL who have received appropriate interventions within the first six months of life) as one of three tracer indicators for global surveillance and monitoring of progress in ear and hearing care [2]. In light of this, the World Report on Hearing calls for a 20% increase in effective NIHS coverage by 2030 as one of the targets for scaling up IPC-EHC services. Specifically, countries with less than 50% coverage should aim for at least 50% coverage, countries with 50–80% coverage should aim for a 20% relative increase, countries with coverage rates above 80% should aim for universal coverage, and countries with population groups covered by newborn hearing screening should aim for 95% or greater coverage [2]. The World Report on Hearing also noted that a low- to middle-income country would have a potential return of 1.67 international dollars for every dollar invested in NHS and a high-income country would have a return of 6.53 international dollars. In addition, the lifetime value of DALYs avoided (disability-adjusted life years; a measure that combines the number of years of life lost to premature death with the loss of life due to the severity of a disability or disease) would be 21,266 international dollars per person in low- and middle-income countries, and as much as 523,251 international dollars for high income settings [2].

This article reviews the global status of newborn and infant hearing screening as assessed by the above-mentioned survey [33] and the effectiveness of NIHS in early detection of and intervention for PCHL in light of several recent WHO publications, particularly the World Report on Hearing [2] and the handbook Hearing Screening—Considerations for Implementation [1].

## 2. Materials and Methods

The World Report on Hearing [2], among others, refers to the NIHS global status survey [33], which was based on a 19-item questionnaire examining the country-specific NIHS status for 158 countries. The questionnaire is attached as Supplementary Materials to this article. It collected information for a reference year on (1) the proportion of infants in the number of live births who participated in NHS or hearing screening in the first year of life; (2) whether the hearing screening program was designed to include all infants born in a nation, state, region or hospital (universal screening) or only infants at risk for PCHL (targeted screening); (3) the screening methods used: otoacoustic emissions (OAE) or automated brainstem audiometry (AABR) alone or two-stage OAE-AABR screening in which AABR is performed if a baby failed OAE screening, questionnaire-based screening or other procedures; (4) the proportion of all and screened infants who required audiologic diagnosis because they were suspected of having a hearing loss and the proportion of the two groups of infants in whom such diagnosis was made; (5) the prevalence of early PCHL per 1000 infants; (6) the proportion of hearing-impaired infants identified by hearing screening; (7) the mean or median age and age ranges of both diagnosis and treatment initiation for screened and unscreened hearing-impaired infants; (8) the proportion of screened and unscreened hearing-impaired infants who required and received immediate treatment and the proportion of all and of screened hearing-impaired infants with treatment initiation before six months of age; (9) whether and when a country’s government mandated hearing screening; (10) the type of screening mandated; (11) the location of screening and the type of screening personnel; and (12) the proportion of birth facilities in a country that implemented NIHS programs. PCHL was defined as a permanent hearing loss of >20 dB HL (decibel hearing loss) in the better hearing ear for bilateral hearing loss or in the worse ear for unilateral hearing loss, averaged over the frequencies 0.5, 1, 2, and 4 kHz. This is in line with the new classification published in the World Report on Hearing for binaural hearing loss and even falls below the threshold for unilateral hearing loss given in that report [2].

Distribution of questionnaires via e-mail started in 2014; updates were accepted until 2019. The original reference year was 2014, but only older data were available for many NIHS programs, so the survey period for the final data ranged from 2009 to 2019. The survey began by identifying individuals in as many countries as possible who were involved in ear and hearing health and who were able to provide information on the status of NIHS in their country. It took substantial effort to identify such key people, especially in regions where audiology services were scarce. As a result, this process took years for some countries, particularly in Africa, Latin America, and parts of Asia. The identification of key individuals was supported by various organizations related to ear and hearing care such as the International Society of Audiology (ISA), Hearing International, the Coalition for Global Hearing Health (CGHH), the International Association of Logopedics and Phoniatrics (IALP), the International Working Group on Childhood Hearing, the American Academy of Audiology (AAA), but also by non-governmental organizations (NGOs) like Soundseekers and the Christoffel Blindenmission (CBM) and in French-speaking Africa through the network of the Société Oto-rhino-laryngologie (ORL) des pays francophones d’Afrique (SORLAF), either by signing a letter of invitation or by contact referral or by direct data delivery.

Many individuals were identified through personal contacts of the authors of this article through conferences, through contacts via the WHO Programme of Prevention of Deafness and Hearing Loss, others through national or state NHS centers, ministries of health, regional WHO offices or through authors of publications with pediatric-audiological topics. Inquiries were made via e-mail, telephone or personal contact by the first author. Various contacts also passed the questionnaires on to other colleagues able to provide information. Whenever possible, the data were checked for plausibility and confirmed by either a second or third independent person or by one of the named institutions.

## 3. Results

**Figure 1:**
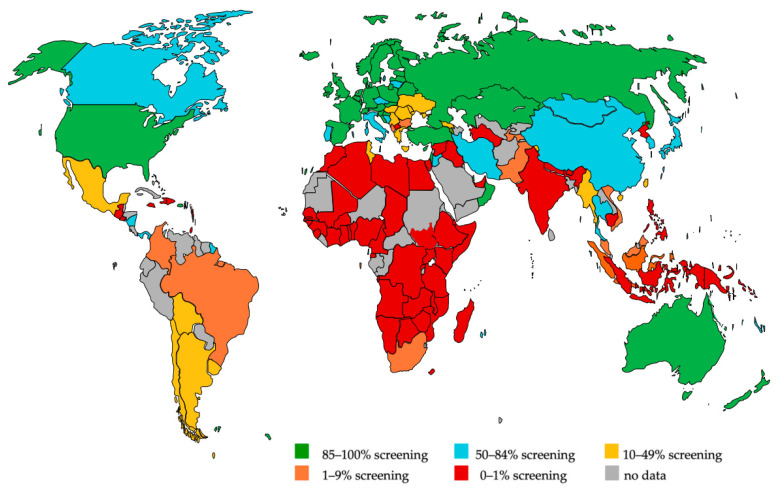
Country-specific coverage of newborn and infant hearing screening programs (modified from [33] with permission).

The survey provided information from 158 countries. The results indicated that less than one-third of the world’s newborns and infants were enrolled in universal NIHS programs covering at least 85% of all babies in a region or country, despite evidence of the effectiveness of this strategy for optimal rehabilitation of deaf and hearing-impaired children. In contrast, about 38% of babies are born in countries with no or minimal NIHS of less than 1% coverage (Figure 1). The countries that completed the survey represent almost 95% of the world’s population [33]. The prevalence for infant PCHL identified through NIHS programs ranged from 0.3–15.0 per 1000 infants with a median of 1.70, according to survey results [33]. This figure approaches WHO prevalence estimates of 2 per 1000 for the neonatal period [2].

According to the survey, most NIHS programs use “physiological” (objective) screening methods. These include otoacoustic emissions (OAE) measurements to assess inner ear function, automated auditory brainstem response (AABR) recordings to evaluate auditory pathway function up to the brainstem, and two-stage OAE-AABR procedures, i.e., AABR is recorded only when OAE fails. These methods achieve high validity, in contrast to behavioral or questionnaire-based methods [34]. Of the infants assessed with these standard methods in a reference year, 66.5% were assessed with OAE alone, 14.3% with AABR alone, and 19.2% with an OAE-AABR combination. Only six countries reported using behavioral methods, and maternal questionnaires or tympanometry were rarely employed. OAE was the preferred method in 57% of countries, followed by OAE-AABR (30%) and AABR (11%) [33].

**Figure 2:**
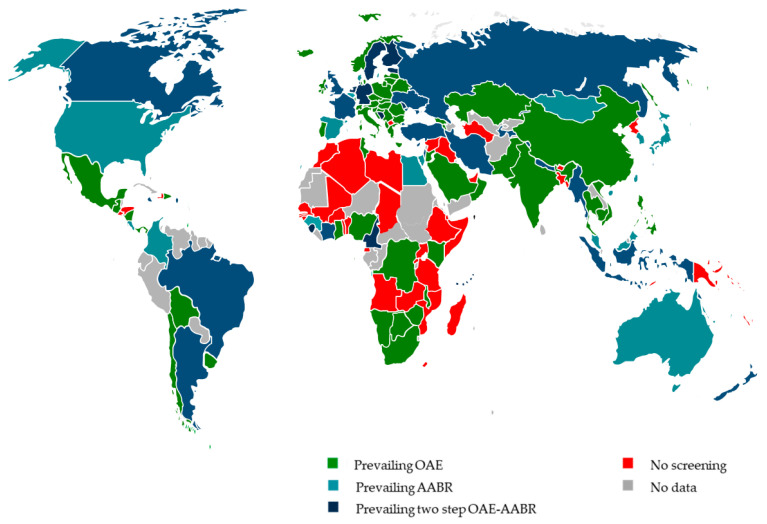
Prevailing screening method used for NHS by country.

Figure 2 shows the methods predominantly used in each country. It should be noted that in many countries only minimal, mostly hospital-based screening was reported. The predominant screening method shown in Figure 2 for these countries may give the somewhat misleading impression that this method is used throughout the country. Therefore, Figure 2 must be viewed in the context of Figure 1. Countries such as Algeria, where screening has only been done in studies, are marked as “no data available” because the studies do not reflect the everyday situation. It should also be noted that in some countries there is a near balance between the method shown in Figure 2 and another screening method.

Infants with a PCHL who underwent hearing screening were diagnosed as hearing impaired at an average age of 4.6 months and received initial treatment at an average age of 6.7 months. This was one of the most encouraging results of our study because it demonstrates that treatment begins at an age that still falls within the sensitive periods of basal maturation of the neural structures of the auditory pathway (e.g., sprouting of dendrites and spines, synaptogenesis, contact stabilization, and breakdown of unneeded connections), which includes a cascading sequence of opening and closing developmental windows shortly after hearing onset, during which the infant brain is still well amenable to treatment [35,36]. This was not the case in the unscreened children, who were diagnosed with hearing loss at an average age of 34.9 months and did not receive initial intervention until 36.7 months of age.

On average, 4.5% of the babies who underwent NIHS failed the screen. The proportion of infants failing screening was significantly lower in countries with high NIHS coverage of 85% or more and its range was narrower (0.3–11.6), compared to countries with lower screening coverage. It is particularly concerning that 17.2% of children who failed screening were lost-to-follow-up. These rates were 7% lower in countries with high NIHS than in countries with low coverage [33].

The lack of systematic data collection and databases compromises the quality of many screening programs. Survey results showed a lack of tracking programs for babies who did not receive screening and for those who failed screening and would need to be referred to audiological diagnostic and treatment services. Without tracking, the lost-to-follow-up rate is generally high or simply unknown. This can also be seen in the wide range and many apparently unreasonable figures for reported lost-to-follow-up rates, which ranged from 0% to 98.2% of infants who failed screening, depending on the program [33].

It is striking that screening coverage and other measures are closely associated with average living standards, as measured by national average nominal gross domestic product per capita (GDP). Countries with NIHS coverage of 85% or more have median living standards that are 10 times higher than those in countries with screening coverage of less than 10%. However, countries with relatively high coverage have large variance in GDP and include countries with low GDP of <10 (e.g., Belarus, China, Kazakhstan, Marshall Islands, Micronesia, Russia). GDP correlates negatively with screening failure rate, prevalence of PCHL, median age at diagnosis, and median age at intervention onset. The dramatically lower standard of living of countries with low screening coverage is more significant given that 80% of people with disabling hearing loss live in low- and middle-income countries [37], where poor birth conditions and lack of vaccination programs contribute significantly to the incidence of PCHL [38], and global production of hearing aids meets less than 3% of the needs of these countries [39]. A NIHS with coverage of 85% or more has been achieved in countries such as the United States, Uruguay, most European countries, Israel, Kazakhstan, Oman, Qatar, South Korea, the Seychelles, Australia, New Zealand, and Pacific Island nations that are territories of the United States. Other countries such as Canada, Mongolia, Panama, and China have implemented large-scale NIHS programs, although they have not yet achieved nationwide coverage. Interestingly, these countries are by no means all high-income countries. Thus, implementation of NIHS programs appears to depend not only on national wealth, but also on other factors such as awareness and attention to infant hearing health among a country’s policymakers and health professionals. This is also consistent with the fact that although national mandates were associated with screening coverage (rho = 0.51), such mandates do not appear to be essential, as nine of the 38 countries with high NIHS coverage had no mandate [33].

Hearing screening was performed in birth facilities in 93% of the countries, in other places such as pediatric, hearing care, immunization or well-baby clinics in 51% and in the homes in 14% of the countries (percentages sum to >100% because a single country could have screening done in multiple places). It was carried out by physicians (26% of the countries), audiologists, audiological staff, or technicians (69% and 16%, respectively), nurses, midwives and nonprofessionals such as community health workers (69% and 24%, respectively) [33].

## 4. Discussion

As mentioned in the World Report on Hearing, hearing screenings can follow one of two approaches: They may be universal, aiming to cover all infants in a country or region, or they may be targeted to infants at risk for early hearing loss, which has been reported to affect approximately 8% to 10% of all newborns [40]. However, it has been demonstrated that targeted screening would miss approximately 40% to 50% of all infants with hearing loss because they do not have risk factors for early hearing loss [40]. Screening can also be opportunistic [2], such as when parents suspect hearing loss in their child and bring their child for hearing screening. According to our survey there are relatively many countries with screening coverage of about 1% where infants are apparently only screened at the request and often at the expense of parents, usually limited to individual hospitals [33]. As the survey also demonstrated, universal approaches are beginning to be implemented in many countries. However, because it takes a great deal of effort and resources to implement a NIHS for an entire country, state, or region, targeted, opportunistic, or hospital-based solutions exist alongside universal or regional approaches [33]. Meanwhile, a large population-based study of long-term outcomes of children with PCHL comparing the three screening approaches (UNHS, “at-risk” screening, and opportunistic screening) demonstrated the clear advantages of universal screening in terms of age at diagnosis of PCHL, receptive and expressive language, and receptive vocabulary development over the other two types of screening [41]. Therefore, the UNHS approach is preferred, as proposed by WHO [1,2] and supported by our survey [33] and other studies [42].

A key issue to improve the effectiveness of NIHS programs worldwide and thus its cost-efficiency is the reduction of high lost-to-follow-up rates. In our survey, from the 27 countries that provided trustworthy data nearly half (48%) exceeded 30% of lost-to-follow-up cases and thus failed the minimum of 70% return-for-follow-up suggested by the Joint Committee on Infant Hearing (JCIH) [43,44] and others [45]. This is consistent with the results of a meta-analysis by Bussé et al., in which lost-to-follow-up rates were above the threshold of 30% in 18 of 41 studies (44%), implying that nearly half of NIHS programs are not completing an audiological diagnosis for many children with suspected hearing loss. In particular, countries with poorly functioning screening programs tend to have high lost-to-follow-up rates [46]. Our survey also showed a 7% higher lost-to-follow-up rate of countries with low screening coverage compared to countries with well-functioning UNHS programs [33]. Reasons for inefficiency of follow-up procedures include lack of data collection and tracking systems and audiology services, educational disparities, and lack of knowledge among parents concerning hearing loss [47]. In addition, distance to the hospital or transportation difficulties, unspecific parental concerns and anxiety, procedural problems, and inadequate availability and visibility of services have been identified by the WHO as factors preventing follow-up consultations [39]. One of the most efficient strategies to overcome these hazards is the installation of appropriate data management systems [39,47]. Other solutions, in particular for resource-limited settings, may include community based EHC services and the integration of follow-up services with child vaccination programs [48,49]. WHO has also recommended the establishment of national committees for ear and hearing care. Their tasks include the central coordination, quality assurance, and effectiveness monitoring of their national NHS programs and thus the containment of return-to-follow-up-reducing factors [2].

The prevalence numbers of PCHL in our survey ranged from 0.3 to 15 per 1000 newborns with a median of 1.70 [33]. This agrees well with the results of a recent systematic review and meta-analysis, which reported an overall prevalence of 2.21 per 1000 (range 1–6) [46]. The World Report on Hearing includes a new classification of hearing loss, which by definition starts at a mean hearing loss of >20 dB HL and comprises unilateral hearing loss. This will elevate prevalence numbers in the future and further challenge hearing screening programs to focus more on mild and unilateral hearing loss [50].

Genetic factors account for about 50% of neonatal hearing loss [51] and syndromic and non-syndromic hearing disorders are associated with over 250 genes that are inherited in an autosomal dominant, autosomal recessive or X-linked manner [2]. Syndromic hearing loss is often associated with additional visual, neurologic, endocrinologic, or other disorders. Our survey documents the highest prevalence numbers in countries with high proportions of inherited forms of sensorineural hearing loss associated with higher rates of consanguineous marriages such as Pakistan, Egypt, Algeria, Jordan, and Turkey [33]. This fits with findings from the UK Millennium Cohort Study, which identified an increased risk of PCHL for children of, among others, Pakistani and Bangladeshi descent [52].

## 5. Conclusions

To further expand and improve NIHS programs around the world, the following initiatives should be considered following the results of our survey and the WHO recommendations [1,2,3,33,34,53].

- Governments should take on leadership responsibility with regard to the strategic direction and implementation of measures to address hearing loss in an integrative way in their health systems [2,30,32]. That this has not happened sufficiently in the past [31] is testament to the international lack of government governance and leadership in ear and hearing care.
- Governments should establish national committees on ear and hearing care, led by their ministries of health and a national ear and hearing care coordinator to develop EHC strategies that include national NHS programs as an important component [54,55]. Committees should be multi-professional in composition and include all professional and stakeholder groups involved with EHC.
- Governments should take advantage of assistance offered by WHO in planning and implementation of national EHC strategies and explicitly on the implementation, organization, control, and monitoring of NHS programs. Currently available documents include the World Report on Hearing [2], an Ear and Hearing Care Situation Analysis Tool [53] a Planning & Monitoring of National Strategies for Ear and Hearing Care Manual [55], the handbook Hearing Screening—Considerations for Implementation [1], and others [34].
- The WHO has identified raising public awareness and reaching policymakers, health professionals and stakeholders as critical to implementing and funding national EHC policies. National committees that centrally coordinate strategic tools and information materials should be represented in initiatives related to technology, training, infrastructure, equipment, finance, and advocacy. Standard operating procedures (SOPs) should unify workflows, criteria, and benchmarks, e.g., the allowable repeats of a failed primary hearing screenings and how, where, and when positively screened babies should be followed up [53,55]. Regular data analysis, e.g., on coverage and failure rates of screening, should serve as process indicators of quality control of NIHS programs [55].
- Governments should enact legislation to formalize the operation of NIHS programs.
- Data collection and tracking systems should be established, preferably from the beginning of NIHS program implementation, to track babies who have failed or missed screening and to provide systematic information on the coverage and quality of screening, intervention, and rehabilitation services for individual children and their families, as well as on successes and gaps in these services. These systems should consider the use of telemedicine components and bidirectional data flow between decentralized screening devices and hearing screening centers [12].
- NIHS programs should include the opportunity for case discussions in professional excellence circles with boards of experts [54].
- Governments should guarantee equal access to pediatric audiology services for all infants with PCHL. This requires pursuing creative and affordable solutions for wider availability of hearing technology including bulk purchases of hearing aids or implants [29,30,54].
- As with other diseases, hearing loss rehabilitation is underrepresented in many countries. Therefore, a new WHO resource currently being developed in collaboration with Cochrane as Package of Rehabilitation Interventions (PRI)—a set of prioritized evidence-based interventions along with resource requirements for their implementation—also focuses on hearing loss rehabilitation [56]. Regarding PCHL, it is therefore important that professionals in EHC are aware of these evidence-based interventions and that they are made available to more and more children [57].
- Countries with high NIHS coverage should ensure equitable access in their population. They should volunteer to be available as a resource to countries with more limited NIHS programs.
- In addition to NIHS programs, measures to prevent neonatal hearing loss as described in a WHO publication on childhood hearing loss [3] and in the World Report on Hearing [2] need to be installed and must take the demographic profile and resources of a country into account. They include, for example, preventing CMV infections in pregnancy through hygienic measures, restricting the administration of ototoxic drugs in the neonatal period, or improving neonatal care for premature infants. Identification of the cause or risk for early childhood hearing loss should be encouraged, as it may influence further action [58]. For example, diagnosis of congenital CMV infection should be made possible shortly after birth, e.g., by screening, to determine whether the infection is prenatal or postnatal. Congenital CMV infections result in late-onset hearing loss in about 50% of infected children that would escape NHS [59], whereas postnatal infections do not compromise a child’s hearing. Knowing that hearing loss is genetically caused could change a family’s decision making regarding interventions and further family planning. Especially given that consanguineous parents are at increased risk for congenital health problems in their children, such as hearing loss [60,61,62,63,64], and consanguineous marriages account for approximately 20–50% of marriages in some parts of the world [2], genetic counselling in a non-directive manner and education can be preventive [2,51,55,65].
- NIHS followed by early intervention and accompanied by measures preventing childhood hearing loss has been shown to be effective, cost efficient, and an excellent investment of resources. Our survey confirmed for the first time worldwide that newborn hearing screening benefits children in terms of early diagnosis and treatment initiation of PCHL, but that large global disparities remain. As the world moves toward achieving the 2015 Sustainable Development Goals and universal health coverage, it is important that the needs of children with hearing loss are addressed and “no one is left behind”. For them all to achieve their “highest attainable standard of health”, the provision of NIHS as part of national plans for universal health coverage is a matter of equity and equality.

## Data Availability

The data presented in this study are available on request from the corresponding author. They are not publicly available to avoid misunderstandings because in many cases they come from sources that are not formally confirmed.

## Supplementary Materials

The following are available online at https://www.mdpi.com/article/10.3390/jcm11010271/s1, Questionnaire: Survey on the international status of early hearing detection and intervention. international survey on newborn and infant hearing screening.
