# Supplementary material for: Newborn Hearing Screening Benefits Children, But Global Disparities Persist
Source: J Clin Med. 2022 Jan 5;11(1):271. doi: 10.3390/jcm11010271 (PMC8746089; doi:10.3390/jcm11010271)
Supplement: Supplementary file 1 [file jcm-11-00271-s001.zip › jcm-1515837-supplementary.pdf]

SURVEY ON THE INTERNATIONAL STATUS OF EARLY HEARING DETECTION AND INTERVENTION

INTERNATIONAL SURVEY ON NEWBORN AND INFANT HEARING SCREENING

Country of report: ____

The reporting year is one of the years 2013, 2014 or 2015 (choose one year among the three given years where you have the most complete data or best estimates).
Reporting year: 20__

(1) Please indicate the main source(s) of your data (for example birth registry; national institution of statistics):
____

(2) Number of live births (alive after 4 days of life) in the reporting year:_______ Data source(s): ☐ Act. ☐ Est.

(3) Percentage of babies or infants born in the reporting year who underwent a hearing screening ☐ Act. ☐ Est.
At all __% of _______(number of life births)
Universal Newborn Hearing Screening (UNHS): __% of _______(number of life births)
Later screening in the 1st year of life: __%
Targeted screening of babies at risk:__% Data source(s):

(4) What method is used to do hearing screening? Data source(s):
OAE alone: __% of _______(number of screened babies) ☐ Act. ☐ Est.
AABR alone: __%
2-stage OAE-AABR screening:__%
(Please be aware: 2-stage means that you at first screen with one method (most often TEOAE), and only in case of a failed screening with another one (most often AABR). If you have such a screening for let´s say 100 percent of babies and 90 pass an OAE screening and do not need an AABR, and the remaining 10% babies undergo an additional AABR-screening, you have a 100% 2-stage OAE-AABR screening and 0% OAE alone screening (and not a 90% OAE alone screening and a 10% 2-stage OAE-AABR screening)).
Questionnaire-based screening: __%
Another screening method: __% Which method?__________

(5) Percentage of infants born in the reporting year who needed a diagnostic audiological evaluation:

__% of ____________(number of born babies) Data source: ☐ Act. ☐ Est.
Percentage of infants born in the reporting year who needed a diagnostic audiological evaluation and underwent a hearing screening from all born babies: __% of ____________(number of born babies) ☐ Act. ☐ Est.

(6) Percentage of infants born in the reporting year who received a diagnostic audiologic evaluation from all born babies: __% of ______(number of born babies) Data source: ☐ Act. ☐ Est.

Percentage of infants born in the reporting year who received a diagnostic audiological evaluation and underwent a hearing screening from all born babies: __% of ____________(number of born babies) ☐ Act. ☐ Est.

(7) Proportion of infants born in the reporting year who were identified with a Permanent Childhood Hearing Loss (PCHL)* from all born babies including late identified cases (=prevalence for a one year cohort):
__per 1000 of ________(number of born babies) Data source: ☐ Act. ☐ Est.

(8) Percentage of infants born in the reporting year and identified with a PCHL who underwent a hearing screening
__% of ________(number of babies with PCHL) Data source: ☐ Act. ☐ Est.

(9) Median (or Mean) age and age range of diagnosis for hearing impaired children who underwent a hearing screening:
a) Median age (months):____ (Mean age:___)
b) Minimum age (months):
c) Maximum age (months): Data source:

(10) Median (or Mean) age and range of diagnosis for hearing impaired children who did not undergo a hearing screening before they were diagnosed with a PCHL:
a) Median age (months):____ (Mean age:___)
b) Minimum age (months):
c) Maximum age (months): Data source:

(11) Median (or Mean) age and range of hearing impaired children who underwent a hearing screening at start of treatment:
a) Median age (months):____ (Mean age:___)
b) Minimum age (months):

c) Maximum age (months): Data source:

(12) Median (or Mean) age and range of hearing impaired children who did not undergo a hearing screening at start of treatment:
a) Median age (months):____ (average age:___)
b) Minimum age (months): c) Maximum age (months): Data source:

*PCHL includes unilateral or bilateral permanent hearing loss, which has been confirmed through a battery of audiometric tests that result in hearing loss detection at the better ear and averaged over frequencies 0.5, 1, 2, and 4 kHz greater than 20 dB HL.
**Intervention may include (but is not limited to) fitting with hearing devices, speech-language therapy, early intervention programing by a parent-infant specialist, medical or surgical treatment, etc. In cases where it is unclear whether treatment is required, further monitoring also counts as intervention.

(13) Percentage of infants with PCHL born in the reporting year who needed intervention** from all babies diagnosed with a PCHL:__% of ________(number of babies with PCHL) Data source: ☐ Act. ☐ Est.

(14) Percentage of infants with PCHL born in the reporting year who received intervention*** from all babies diagnosed with a PCHL:__% of ________(number of babies with PCHL)* Data source: ☐ Act. ☐ Est.

Percentage of infants with PCHL born in the reporting year receiving intervention who underwent a hearing screening from all babies receiving intervention:___% of _______(number of babies receiving intervention) ☐ Act. ☐ Est.

(15) Percentage of infants with PHCL born in the reporting year who received intervention before 6 months of age from all babies receiving intervention:____% of _______(number of babies who received intervention) Data source: ☐ Act. ☐ Est.

Percentage of babies with PCHL born in the reporting year and received intervention before age 6 mos. who underwent a hearing screening from all babies receiving intervention: __% of _____(number of babies receiving intervention) ☐ Act. ☐ Est.

(17) Check which if any of the following hearing screenings are mandated by law in your country:

Newborns ☐ Later in the 1st year ☐ At-risk ☐
If yes, please specify when the legislation passed: ____(year)

(18) Give a percentage of places where the screening is done from all screening places.
Birthing facilities: __% Home: __% Other outpatient places: __% Which place?_______

(19) Give a percentage of professionals who perform the hearing screening from all persons who perform the hearing screening.
Physicians: __% Audiologists/audiological staff: __% Nurses: __% Midwifes: __%
Community health workers: __% Others: __% (please specify __________)

(20) Percentage of birthing facilities in your country performing a hearing screening from all birth facilities:
__% of _______(number of birthing facilities) Data source: ☐ Act. ☐ Est.
